# Motor and Sensory Benefits of Mirror Therapy in Children and Adolescents with Unilateral Cerebral Palsy: A Systematic Review and Meta-Analysis

**DOI:** 10.3390/healthcare13131538

**Published:** 2025-06-27

**Authors:** Anna Ortega-Martínez, Rocío Palomo-Carrión, Andoni Carrasco-Uribarren, Marta Amor-Barbosa, Georgina Domènech-Garcia, Mª Caritat Bagur-Calafat

**Affiliations:** 1 Physiotherapy Department, Faculty of Medicine and Health Sciences, Universitat Internacional de Catalunya, Sant Cugat del Vallès, 08195 Barcelona, Spain; aortegam@uic.es (A.O.-M.); acarrasco@uic.es (A.C.-U.); gdomenech@uic.es (G.D.-G.); cbagur@uic.es (M.C.B.-C.); 2 Physiotherapy Department, Fundació Aspace Catalunya, C/Tres Pins 29, 08038 Barcelona, Spain; 3 Physiotherapy Department, Universidad de Castilla-La Mancha, 45071 Toledo, Spain

**Keywords:** unilateral cerebral palsy, mirror therapy, upper limb impairments, hand function, motor impairment, somatosensory impaiment, systematic review, meta-analysis

## Abstract

**Background:** Mirror therapy (MT) creates a cerebral illusion of a normal movement in a paretic limb. Although mirror therapy has been studied as a suitable intervention for children with Unilateral Cerebral Palsy (UCP), a comprehensive understanding of its full range of benefits is still lacking. Thus, the aim of this systematic review and meta-analysis was to determine all motor and sensory effects of MT in children and adolescents with UCP. **Methods:** Clinical trials focused on the application of MT in the upper limb (UL) of children and adolescents with UCP were included. A search was performed in PubMed, Cochrane Library, Web of Science, and LILACS databases. Eleven studies were included in this systematic review. The PEDro scale and the MINORS scale were applied to evaluate the methodological quality of randomized and non-randomized controlled trials, respectively. The Risk of Bias tool was also employed to evaluate the potential bias. In addition, the TIDieR checklist was used to assess the quality of intervention reporting. A random-effects model was used for the meta-analysis. **Results:** The studies included children with UCP from three to eighteen years, classified in Manual Ability Classification System levels I–IV. Motor effects of MT were found in nine studies. Also, two studies reported sensory effects on registration, perception, and proprioception abilities. Qualitative and quantitative analysis showed that MT improved manual dexterity and tactile registration in children and adolescents with UCP. **Conclusions:** MT is a therapy capable of inducing motor and sensory improvements in the affected UL of children with UCP.

## 1. Introduction

Cerebral Palsy (CP) is “a group of permanent neurologic disorders involving movement and posture that result in limited activity and function caused by non-progressive lesions of the developing brain”. Among its subtypes, Unilateral Cerebral Palsy (UCP) refers to cases of CP where the brain lesion impacts both motor and sensory capacities of the contralateral side of the body [1]. The brain lesions are most commonly located in the white or gray matter [2,3,4].

As a consequence of the early brain injury, the developing brain undergoes a process of cortical reorganization, where the brain attempts to adapt by modifying its structural and functional connectivity. The type of lesion, its location, and extent influence the brain’s capacity to reorganize [5,6].

The upper limb (UL) is usually involved, but the extent of the impairment includes several types according to the Manual Ability Classification System (MACS). Frequently, children with UCP are classified in MACS levels I to III [7]. That means that they show limited hand use during bimanual activities and usually need support from their caregivers. Motor impairments directly impact their performance in daily life activities, where the coordination and collaboration of both hands are usually needed [8,9,10,11]. Those children with higher bimanual performance and capacities are likely to show better performance in self-care [12].

Furthermore, limitations in hand use extent negatively impact the quality of life of children and adolescents with UCP. School and daily activities, strength during activities, and physical health are among the main areas of quality of life affected by difficulties in hand performance [13].

Although motor impairments have been widely described as the main causes of difficulties in children with UCP, somatosensory impairments also need to be considered. They may impact the daily life of children with CP, as they are present in 70% of this population [14]. Children with less tactile registration and perception capacities are likely to suffer from more difficulties in the unimanual capacity of the affected upper limb and, subsequently, in bimanual performance. At least 30% of the variance in unimanual capacity and bimanual performance of these children can be a result of a perception impairment [15].

Even though therapies have traditionally been focused on motor impairments, increasing evidence highlights the need to consider sensory impairments, as they may influence and condition motor abilities [16]. Among the most utilized therapies in children with UCP, modified Constraint-Induced Movement Therapy (mCIMT) and Hand–Arm Bimanual Intensive Training have been the most studied ones, with excellent results in motor outcomes [17]. However, effects on sensory outcomes have not been demonstrated [18].

Mirror therapy (MT) has emerged as a promising therapy for children with UCP. First described in 1995, MT consists of placing a mirror between both hands while moving them symmetrically, so that the vision of the non-affected UL is superimposed on the affected UL [19]. Its positive results in adult stroke patients have resulted in MT being adapted to children with UCP, given the similarities in both neurological conditions. In adults, MT improves motor function, the activities of daily living performance, pain, and visuospatial neglect, among other effects [20]. In children with UCP, it has been demonstrated that MT is capable of modulating brain activation patterns, supporting its potential as a plasticity-based therapy [21,22,23].

Despite the fact that the number of studies proposing MT protocols in children with UCP has been rising over the last years, findings remain heterogeneous [24]. Nevertheless, it is one of the few therapies that has been suggested as capable of improving somatosensory registration and perception [25]. A comprehensive synthesis of the effects of MT is still required in order to better understand the areas where it could have a positive impact. Moreover, the fact that MT is an easy, cost-effective therapy with no risk of adverse effects highlights the need for a more comprehensive understanding of its possible benefits [18]. Therefore, the aim of this systematic review and meta-analysis is to determine the motor and sensory effects of MT in the affected UL of children with UCP.

## 2. Materials and Methods

### 2.1. Study Design

The present study is a systematic review and meta-analysis. The study protocol was registered in the International Prospective Register of Systematic Reviews (CRD42022372557). No deviations from the registered protocol were made. This study was reported according to the Preferred Reporting Items for Systematic Reviews and Meta-analysis statement and the Cochrane recommendations for systematic reviews [26,27].

### 2.2. Search Strategy

For this systematic review, different databases were consulted: PubMed, Cochrane Library, Web of Science, and Latin American and Caribbean Health Science Literature. We also conducted a cross-reference search to identify other potentially relevant references. A comprehensive literature search was performed covering the period from inception to April 2025. The search strategy included terms related to the population [(“unilateral spastic cerebral palsy” OR “unilateral cerebral palsy” OR “hemiplegic cerebral palsy” OR “hemiplegia”)] NOT stroke. For the intervention, other terms were used (“mirror therapy” OR “mirror box” OR “motor imagery” OR “mirror movement therapy”). Regarding the outcome, we used different terms that define motor and sensory functions of the hand (“bimanual performance” OR “somatosensory function” OR “position” OR “strength” OR “gross motor skills” OR “fine motor skills” OR “unimanual capacity”). All terms were connected with Boolean operators adapted to each database. Supplementary Materials contain further details of the search strategy (see S1).

### 2.3. Eligibility Criteria and Study Selection

This systematic review and meta-analysis included clinical trials (randomized clinical trials, controlled clinical trials, and crossover studies). Thus, any other type of study design was excluded from the study selection. The population to whom this review is addressed is children and adolescents with UCP. That means that only studies focusing on children or adolescents from 0 to 18 years with UCP were included, excluding those studies that focused on adult population or children without a congenital cerebral lesion. Studies were only included if the intervention consisted of MT, administered to the upper limb. Therefore, we excluded studies if (a) they did not focus on MT; (b) MT exercises were not performed bimanually and symmetrically; (c) the intervention targeted the lower limb; or (d) the control group did not consist exclusively of children and adolescents with UCP. Moreover, only interventions comprising at least two sessions were considered to be eligible. Regarding the outcomes, those studies that assessed any motor or sensory measurements with valid and reliable tools were considered to be eligible for this review. Therefore, the included studies could assess (a) bimanual performance and/or unimanual capacity, with questionnaires or scales; (b) gross or fine motor skills, with tests, scales, or dynamometers; (c) sensory impairments, with tests or sensory instruments. We gathered outcome data changes from baseline, post-intervention, and follow-up assessments, when available.

Although no study design filters were used during the database search, only randomized controlled trials, non-randomized controlled trials, and crossover trials were included in this review. We applied these criteria during the screening and selection process. No restrictions were applied regarding language or year of publication.

### 2.4. Data Extraction

After eliminating the duplicates, R.P.-C. and A.O.-M. independently revised the titles and abstracts found with the search strategy, following the criteria described above. M.C.B.-C. acted as arbitrator when discrepancies occurred at this point. After that, full text of the rest was read in order to decide the relevance of each of the studies. A final consensus meeting was held to determine the eligibility of the articles in which there were discrepancies. In addition, we also performed a cross-reference search to identify other potentially eligible studies.

The analyzed data available in this review was extracted from text, tables, and figures given in the studies. The extracted data included the following: (1) characteristics of the population; (2) description of the interventions; (3) outcome measures and measurement tools; and (4) results. Intervention details were extracted by using the “Template for Intervention Description and Replication” (TIDieR), as it was also used for describing the quality of the reported interventions [28].

Qualitative analysis was performed for all studies. Moreover, quantitative synthesis was performed when possible.

### 2.5. Quality of the Evidence and Risk of Bias

As for the data extraction, A.O.-M. and M.A.-B. independently assessed both methodological quality and the potential risk of bias of the included studies. M.C.B.-C. acted as arbiter in both processes in a final consensus meeting held to resolve discrepancies.

The methodological quality of the included studies was assessed using the appropriate tools for each study design included in this review: the PEDro scale [29,30,31] and the MINORS tool [32]. The PEDro scale was used for assessing the quality of all studies included in this systematic review. A score of 4 or less was considered poor quality, 4 to 5 was considered fair, 6 to 8 was considered good quality, and 9 or above was considered high quality. The first item of the PEDro scale was scored, but, as it responds to the external validity of studies and not to their internal validity, it was not included in the total score calculation [31]. Moreover, the MINORS tool was used for non-randomized studies, as it is a valid, reliable, and more suitable tool for non-RCTs due to its methodological characteristics. It comprised a total of 12 items—8 general items for non-randomized studies and 4 additional items for comparative studies. Each item scored 0, 1, or 2, depending on whether it was not reported, reported but inadequate, or reported and adequate, obtaining a total scoring of 16 for non-comparative studies and 24 for comparative studies [32].

Regarding the potential risk of bias, it was assessed by using the Risk of Bias tool (RoB 2). This tool addresses the potential risk of bias by classifying studies as “low risk”, “unclear risk”, or “high risk” [33].

### 2.6. Quality of the Reported Interventions

The TIDieR guideline was used to describe the intervention details given in all studies [28]. We collected information from all studies included in this systematic review regarding the name of the interventions, their key components, required materials, and the providers. Additionally, we gathered details on the timing and setting of the interventions, dosage, tailoring, modifications for participant adaptation, strategies to ensure adherence, and final compliance.

### 2.7. Data Synthesis and Analysis

Data were pooled when at least two studies were deemed comparable, using RevMan 5.4 software. Studies were considered comparable when an intervention of MT was applied and compared to other interventions or bimanual exercises without the mirror. The mean difference (MD) and standard deviation (SD) were used as intragroup effect size measures. For studies that did not report MD and SD but provided sufficient raw data, these values were calculated in accordance with the Cochrane Handbook for Systematic Reviews of Interventions [34]. Between-group comparisons were reported as MD with a 95% confidence interval (95% CI). Statistical significance was set at *p* < 0.05. All data were organized in tables and categorized by intervention type, outcome measures, and results.

A random-effects model was used to conduct the meta-analysis, accounting for the possibility that the included studies were not estimating the same underlying intervention effect [27]. Heterogeneity was assessed by examining the similarity of point estimates, the overlap of confidence intervals, the context of the studies, and the I^2^ statistic reported in the forest plots [35]. To assess publication bias and the influence of individual studies, we visually inspected the forest plots and performed sensitivity analyses by systematically excluding each study. None of the meta-analyses included ≥10 trials, which is the recommended minimum for conducting funnel plot analyses.

## 3. Results

### 3.1. Literature Search and Screening

**Figure 1:**
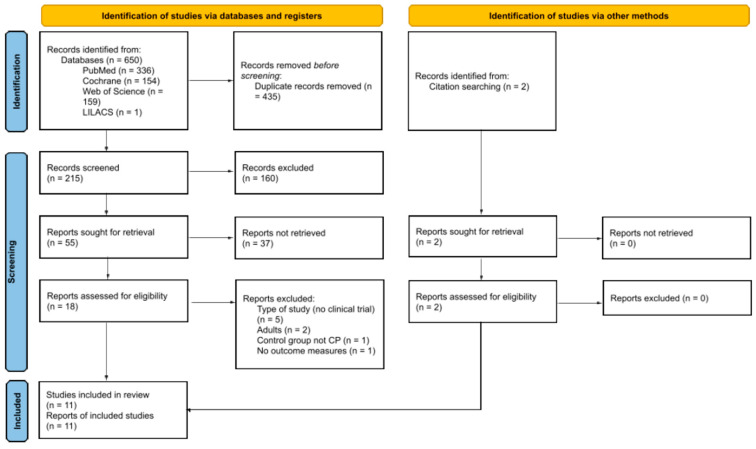
Flow diagram.

Initially, 650 references were identified as a result of the database search process. From those, 435 duplicates were found and, therefore, eliminated from the selection. Titles of the remaining 215 references were analyzed. After that, abstracts of the 55 references were screened. Through that process, we identified 18 studies as eligible for inclusion in this review, all of which we subsequently analyzed in depth. We excluded most studies at this stage due to their study design, as they did not align with the types of studies targeted by this review. Other studies were not included due to population, control, or outcome incompatibilities. As a result of citation searching, two additional articles were identified and incorporated into this review. Thus, 11 studies were finally included in this review. Figure 1 shows more detailed information regarding the selection and screening process.

### 3.2. Characteristics of the Eligible Studies

This systematic review and meta-analysis comprised 11 studies, published between 2011 and 2025. Regarding the study designs, a total of seven studies were RCTs [36,37,38,39,40,41,42], while two were quasi-experimental designs [43,44], and the other two were crossover studies [45,46].

A total of 430 subjects with UCP were included, pooling samples of all the studies, ranging from 3 to 18 years old [36,37,38,39,40,41,42,43,44,45,46]. The majority of the studies included children and adolescents classified in MACS levels I to III [37,38,42,45]. Two studies only included levels II and II [36,40], while another one only included children in level III [39]. The remaining study included levels I to IV [46]. It is notable that this information was not reported in three of the studies [41,43,44]. The number of cases with right hemiplegia was notably higher compared to left hemiplegia—204 cases in front of 93 [37,38,39,40,42,43,45,46]. Three studies did not report the affected side of children included in their research [36,41,44]. The sample sizes of the studies included ranged from 6 to 90. Table 1 shows a brief description of the studies included in this review, by means of year of publication, country, study design, and characteristics of the population.

A highly detailed description of the characteristics of all different MT interventions included in this systematic review can be found in Supplementary Materials (see S2), in the TIDieR checklist [28]. The application of MT differed between all authors. The number of MT sessions ranged between 1 and 60 sessions. As the duration of the sessions was also different between studies, the total dosage of therapy ranged from 90 min to 3600 min [36,37,38,39,40,41,42,43,44,45,46]. Also, the frequency of the MT sessions varied among studies, with variability ranging from one session per week to daily sessions [36,37,38,39,40,41,42,43,44,45,46].

Some authors applied an MT protocol alone, without combining it with any other intervention [43,44,45], while others combined it with a standard rehabilitation program [36,40], a strength training of the upper limb [38], a taping application [40], a neurodevelopment-based program [41], an occupational therapy program [42], or they led the participants to continue with their usual therapies [37,39]. Regarding the exercises included in the MT programs, differences were also found between authors. In 10 of the 11 studies included in this review, bimanual motor exercises were performed [36,37,38,39,40,41,42,43,44,46]. The MT protocol from Auld et al. [45] was the only protocol including a sensory stimulation during the MT, as well as a motor training.

Considering the comparison interventions that underwent controls, it is notable that, while some authors compared MT with the performance of the same exercises in the MT group, without the mirror [37,44,46], the majority used some other therapies. These therapies included mCIMT [36,39,40], bimanual therapy [45], conventional programs [43], occupational therapy [38,42], MT with taping on the upper limb [40], and a neurodevelopment-based program [41].

### 3.3. Methodological Quality and Risk of Bias

The methodological quality of the studies included in this review was analyzed with the PEDro scale [29,30]. Results shown in Table 2 determined the scores obtained by each study. A total mean score of 7.0 was achieved, ranging between 3 and 9. All studies reported the eligibility criteria and “point measures and variability data”. Subsequently, all obtained a score of 1 in these categories. Contrarily, none of the studies reported the blinding of participants or therapists [36,37,38,39,40,41,42,43,44,45,46].

The studies from Elanchezhian et al. [43] and Farzamfar et al. [44] were also assessed with the MINORS scale, as they were non-randomized studies, as shown in Table 3 [32]. Both obtained the full scores in the “prospective collection of data”, “adequate control group”, “contemporary groups”, and “baseline equivalence of groups” items. Their final scores were 16 and 13 out of 24, respectively [43,44].

The risk of bias evaluation was performed by means of the RoB 2 tool [47]. As shown in Table 4, no studies were classified as “low risk”; nine were considered as having some concerns regarding bias [36,37,38,39,40,41,42,45,46], and the other two were considered to have a high risk of bias [43,44]. The most frequent risk of bias was related to “deviations from the intended interventions”, where none of the studies showed a low risk of bias [36,37,38,39,40,41,42,43,44,45,46].

### 3.4. Quality of the Reported Interventions

As previously mentioned, the quality of the reported interventions included in this study was analyzed to evaluate their replicability with the TIDieR checklist [28].

The key components of the experimental and control conditions were described in all studies. However, this was the only category completely described, as the rest of the information was not reported in at least one of the studies [36,37,38,39,40,41,42,43,44,45,46].

With regard to the materials, this information could be inferred from the articles, as the descriptions of the exercises mentioned some material; however, it was not explicitly stated [36,37,38,39,40,41,42,43,44,45,46].

Interventions were provided by a therapist or a researcher in most of the studies [36,37,38,39,40,44,45]. Studies from Elanchezhian et al. [43], Gygax et al. [46], Mohammed et al. [41], and Narimani et al. [42] did not mention who was in charge of the therapies. Additionally, information regarding the time of providing the therapy and/or its setting was missing in several studies [36,38,39,40,41,42,43,44,46]. Two studies failed to include the dosage of the therapies performed by their controls [42,43].

From the remaining categories, Abdel-Ghafar et al. [36] and Elanchezhian et al. [43] were the only ones reporting some information regarding tailoring and/or fidelity. The study from Mohamed et al. [40] was the only one reporting information regarding measures to ensure that the intervention was carried out as planned. None of the studies reported information about adapting or modifying interventions due to participant needs [36,37,38,39,40,41,42,43,44,45,46].

### 3.5. Outcome Measures

As the included studies of this review assessed several motor and sensory components, Table 5 was designed to show all measurements and tools utilized.

From the 11 studies that this systematic review comprises, 8 assessed only the motor components of the affected UL of children with UCP [36,39,40,41,42,43,44,46]. Sensory outcomes were present in two of the studies [37,45], but it was only in that from Auld et al. [45] where somatosensation was the main outcome. Lastly, Kara et al. [38] reported the self-perception of self-care, leisure, and productivity by means of the Canadian Occupational Performance Measure (COPM) [48].

Regarding motor outcomes, grasp strength (GS) [49,50] was the most assessed, as it was present in five studies [36,37,40,42,46]. Secondly, manual dexterity, measured with the Box and Blocks Test (BBT) [51,52], was measured in four studies [36,40,42,44]. Results on the quality of the upper extremity function were shown in three studies [38,39,40], by means of the Quality of Upper Extremity Skills Test (QUEST) [53]. Similar to QUEST assessments, the ABILHAND-Kids scale [54,55,56], the Shriners Hospital Upper Extremity Evaluation (SHUEE) [57], and the Melbourne Assessment 2 (MA2) [58] were utilized by Bruchez et al. [37] and Gygax et al. [46] to assess hand function in different daily activities.

With regard to sensory outcomes, only the tactile registration test, double simultaneous (DS) [59,60], two-point discrimination (2PD) [59,60], single-point localization (SPL) [59,60], and proprioception were found in studies by Auld et al. [45] and Bruchez et al. [37]

In relation to the timing of the evaluations during the studies, only Bruchez et al. [37] and Gygax et al. [46] reported a follow-up assessment at 5 and 3 weeks post-intervention, respectively.

### 3.6. Synthesis of Results

#### 3.6.1. Motor Effects of MT

- Manual dexterity

**Figure 2:**
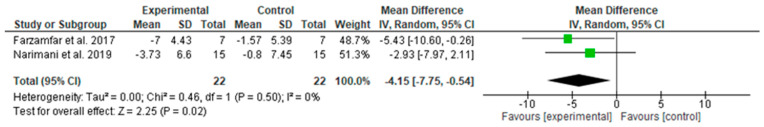
Forest plot of manual dexterity: MT vs. controls [42,44].

Manual dexterity was assessed with BBT in four of the included studies, which were included in the meta-analysis [36,40,42,44]. MT showed better results in manual dexterity, assessed with BBT, compared to control interventions, with statistically significant between-group differences favoring MT (MD = −4.15; 95% CI −7.75, −0.54; two studies, 44 patients) (Figure 2).

The study from Elanchezhian et al. [43] was included in the qualitative synthesis for manual dexterity. It showed an improvement in manual dexterity and was assessed with the Nine-Hole Peg Test [61,62] in the experimental group compared to controls.

**Figure 3:**
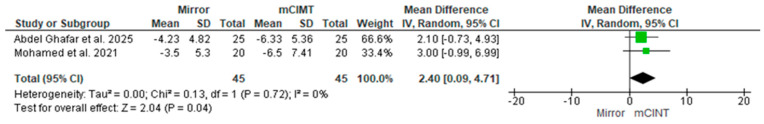
Forest plot of manual dexterity: MT vs. mCIMT [36,40].

When comparing MT versus mCIMT, the latter achieved greater benefits in BBT scores, with statistically significant between-group differences favoring mCIMT (MD = 2.40; 95% CI 0.09, 4.71; two studies, 90 patients) (Figure 3).

- Grasp strength

**Figure 4:**
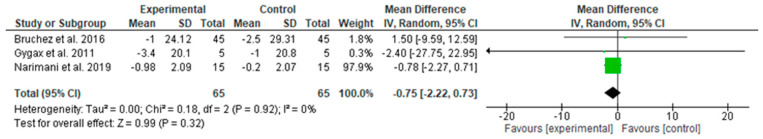
Forest plot of grasp strength: MT vs. controls [37,42,46].

No statistically significant between-group differences were observed in grasp strength between the MT and control groups (MD = −0.75; 95% CI −2.22, 0.73; two studies, 130 patients) (Figure 4).

**Figure 5:**
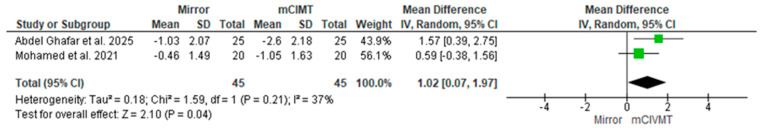
Forest plot of grasp strength: MT vs. mCIMT [36,40].

MCIMT achieved greater enhancement in grasp strength compared to MT, with statistically significant between-group differences favoring mCIMT (MD = 1.02; 95% CI 0.07, 1.97; two studies, 90 patients) (Figure 5).

- Pinch strength

**Figure 6:**
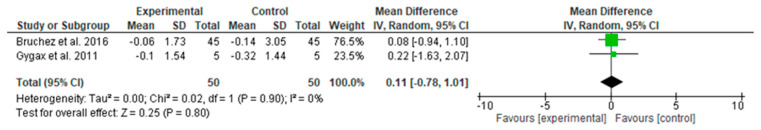
Forest plot of pinch strength [37,46].

Pinch strength [49,50] was measured in two of the included studies, both of them included in the quantitative synthesis of results. No statistically significant between-group differences were observed in pinch strength between MT and control groups (MD = 0.11; 95% CI −0.78, 1.01; two studies, 100 patients) (Figure 6).

- Hand function

**Figure 7:**
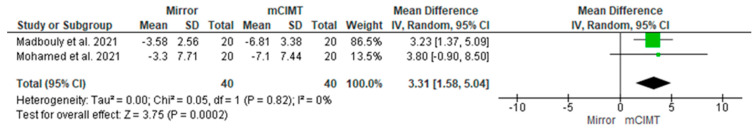
Forest plot of hand function [39,40].

A quantitative analysis was performed with regard to hand function. In that, mCIMT showed better results in hand function, assessed with the QUEST scale [53], compared to MT, with statistically significant between-group differences favoring mCIMT (MD = 1.58; 95% CI 1.58, 5.04; two studies, 80 patients) (Figure 7).

Contrarily, Kara et al. [38] encountered a statistically significant difference (MD = 6.65, *p* < 0.05), with the application of their intervention based on MT and power exercises. It was not included in the meta-analysis due to the combined intervention utilized, as it was significantly different from other studies.

Other studies were included in a qualitative synthesis regarding hand function. While Bruchez et al. [37] did not find a statistically significant improvement in hand function and daily performance with an MT intervention, Gygax et al. [46] encountered improving trends in some subscales of the SHUEE [57].

- Other motor outcomes

Qualitative synthesis was performed for other outcomes assessed in different studies included in this review. Kara et al. [38] showed a significant improvement in the isometric strength of the biceps brachii (MD = 7.11; *p* < 0.05) and triceps brachii (MD = 6.98; *p* < 0.05) in their experimental group (MT + power exercises) when compared to controls receiving occupational therapy.

Elanchezhian et al. [43] showed an improvement in hand function and a reduction in spasticity in their MT group (*p* < 0.05). Similar results were found by Mohammed et al. [41] regarding selective motor control of the upper limb.

#### 3.6.2. Sensory Effects of MT

A qualitative approach was used in order to determine the effects of MT on sensory impairments of children and adolescents with UCP. For Auld et al. [45], MT was capable of improving tactile perception, as children showed a clinically relevant improvement in DS and SPL. For Bruchez et al. [37], this effect was not clear for proprioception and (2PD).

#### 3.6.3. Self-Perception of Daily Performance and Satisfaction

Measurements made by Kara et al. [38] showed that MT led to an improvement in self-perception and satisfaction when assessed with the COPM [48].

## 4. Discussion

This systematic review and meta-analysis aimed to investigate the motor and sensory effects of MT in the upper limb of children and adolescents with UCP. From the initial 650 studies identified as possibly relevant, only 11 met all the inclusion criteria [36,37,38,39,40,41,42,43,44,45,46].

The MT interventions varied greatly in terms of duration, total dosage, and session frequency, leading to high heterogeneity. MT was compared to different conditions: the same exercises as the MT group without the mirror [37,44,46] or other therapies [36,38,39,40,41,42,43,45]. Moreover, the protocols for implementing MT and the information available also differed significantly between authors [36,37,38,39,40,41,42,43,44,45,46].

With regard to the motor effects of the MT, the meta-analyses showed a statistically significant enhancement in manual dexterity when MT is compared to controls performing bimanual exercises without a mirror or with no intervention [42,44]. Contrarily, MT was not effective enough to show better results when compared to mCIMT [36,39,40]. These results differ from the systematic review and meta-analysis by Khan et al. [24], where no statistically significant difference was obtained for manual dexterity. A similar fact occurred with the meta-analysis of hand function. While ours suggested better results with mCIMT than MT, theirs showed slightly better results for MT. Nevertheless, this study did not perform a differentiated meta-analysis comparing MT with mCIMT or controls, and, therefore, it is assumed that results may differ from ours [63]. Also, the study by Mohamed et al. [40] was not included in that review.

Oliva-Sierra et al. [64] concluded that MT could improve UL function, as it increases the muscle strength and manual precision. A similar appreciation was made by Shierk et al. [65], as they suggested that MT could be a promising therapy for the motor function of children with CP. For Yang et al. [63], MT should be the protocol of choice for improving hand function in children with CP. However, our meta-analysis did not find statistically significant differences in terms of its effects on grasp strength [37,42,46] nor pinch strength [37,46]. The study by Kara et al. [38] showed a significant enhancement in hand function. This aligns with Oliva-Sierra et al. [64], but, because Kara et al. [38] combined MT with power exercises in their intervention, their results have to be considered cautiously. Thus, it was not included in our meta-analysis.

Regarding the effects of MT in improving sensory impairments in the upper limb of children with UCP, contradictory results were found. Our results align with other studies where MT seemed to be a promising therapy capable of modulating the somatosensory function [18]. Nevertheless, the studies included in this review did not fully assess tactile registration and perception [59,60,66].

As suggested by Sakzewski et al. [67], therapy dosage may be a crucial factor for its success. The high heterogeneity found in the included studies in this systematic review when considering the implementation of MT may have resulted in insufficient statistical power to detect significant effects. For a better implementation of MT, the setting should also be addressed, as it is stated that children benefit more from home-based interventions than clinical-based ones [17,65]. In this review, the majority of the studies did not report the setting of the intervention [36,38,39,40,41,42,43,44,46]. Furthermore, children with UCP classified in different levels in MACS may benefit from different therapies, as their abilities and performance may differ from their peers [65]. This systematic review has not been able to analyze motor and sensory effects of MT depending on the MACS levels, as this data was not available in the included studies [36,37,38,39,40,41,42,43,44,45,46].

Another possible reason that may have influenced the results found in this review is the employment of multiple different measurement tools. This fact limited the number of studies eligible for inclusion in the meta-analyses performed. Standardizing the evaluation of the main outcomes and employing reliable and valid measurement instruments is essential to ensure consistency and comparability across studies [67].

This systematic review and meta-analysis had some limitations. Firstly, the number of studies that were finally included could have led to some bias, as a growing body of literature could bring more precise results [36,37,38,39,40,41,42,43,44,45,46]. However, we intended to include all available clinical trials in order to present all effects of MT described in the existing literature. Another limitation could be the heterogeneity found in the MT interventions between studies and the tools used to assess motor and sensory components. This fact has limited the comparability between studies when analyzing their results [36,37,38,39,40,41,42,43,44,45,46]. Also, the fact that only two studies reported data regarding sensory outcomes has significantly limited their comparability [37,45]. Moreover, the fact that none of the studies included in this systematic review had a low risk of bias limits the interpretation of the results. Including more studies with a lower risk of bias could enhance the overall quality of the evidence, yielding more reliable and conclusive results. Finally, a limitation in the meta-analyses has been detected, as none of them comprised 10 studies or more. However, we aimed to conduct diverse meta-analyses in order to obtain the most accurate results.

## 5. Conclusions

To our knowledge, this is the first systematic review and meta-analysis to identify all motor and sensory effects of MT on the affected upper limb of children and adolescents with UCP.

MT induces improvements in motor and somatosensory function of the UL, required for a better performance in daily activities. Nonetheless, it is less effective than mCIMT in some motor aspects. Thus, this therapy may be best considered as a complementary option, rather than a standalone intervention, until more conclusive data becomes available. Further robust research comparing its effects on overall manual function, including both motor and sensory aspects, with those of well-established interventions is warranted.

Also, more studies analyzing attributable effects to the protocol of the intervention in terms of dosage, frequency, and setting of the therapy need to be conducted, as well as standardizing protocols of MT to facilitate clinical reproducibility. There is a need for studies to report their interventions more comprehensively and to improve their methodological quality in order to obtain more robust and reliable results. Furthermore, differentiating MT benefits between MACS levels could indicate its best suitability for these children. Finally, further studies using standardized outcome measures are needed to enable meaningful comparisons across trials and to better determine the potential benefits of mirror therapy.

## Figures and Tables

**Table 1:** Description of the included studies and population.

References	Year of Publication	Country	Study Design	Characteristics of the Population						
				n	Age Range	Mean Age (SD)		Affected Side		MACS Levels
						CG	EG	Left	Right	
**Abdel-Ghafar 2025** [36]	2025	Saudi Arabia	RCT	52	5 to 9	6.82 (1.71)	7.46 (1.52)	NR	NR	II–III
**Auld 2017** [45]	2017	Australia	crossover case series	6	6 to 18	10 (median)		3	3	I–III
**Bruchez 2016** [37]	2016	Switzerland	RCT	90	7 to 17	10.8 (4.0)	10.5 (3.0)	38	52	I–III
**Elanchezhian 2019** [43]	2019	India	quasi-experimental	46	3 to 12	7.17 (2.65)	6.96 (1.66)	22	24	NR
**Farzamfar 2017** [44]	2017	Iran	quasi-experimental	14	6 to 12	NR	NR	NR	NR	NR
**Gygax 2011** [46]	2011	Switzerland	crossover	10	6 to 14	NR		4	6	I–IV
**Kara 2020** [38]	2020	Turkey	RCT	34	7 to 16	11.8 (2.85)	12.3 (2.69)	14	16	I–III
**Madbouly 2021** [39]	2021	Egypt	RCT	40	5 to 8	6.5 (1.15)	6.55 (1.14)	0	40	III
**Mohamed 2021** [40]	2021	Saudi Arabia	RCT (3 groups)	60	6 to 9	Group A: 7.6 (0.88) Group B: 7.7 (0.86) Group C: 7.75 (0.91)		6	54	II–III
**Mohammed 2022** [41]	2022	Egypt	RCT	48	6 to 9	6.67 (0.70)	6.80 (0.74)	NR	NR	NR
**Narimani 2019** [42]	2019	Iran	RCT	30	9 to 14	11.30 (1.49)	10.84 (1.62)	13	17	I–III

CG: control group; EG: experimental group; MACS: Manual Ability Classification System; NR: not reported; RCT: randomized controlled trial; and SD: standard deviation.

**Table 2:** PEDro scale scores.

	1. Eligibility Criteria Were Specified	2. Random Allocation	3. Concealed Allocation	4. Groups Similar at Baseline	5. Participant Blinding	6. Therapist Blinding	7. Outcome Assessors Blinding	8. Less Than 15% Drop-Outs	9. Intention-to-Treat Analysis	10. Between-Group Statistical Analysis Comparisons	11. Point Measures and Variability Data	TOTAL
**Abdel-Ghafar 2025** [36]	1	1	1	1	0	0	1	1	0	1	1	8
**Auld 2017** [45]	1	1	1	0	0	0	0	1	0	0	1	5
**Bruchez 2016** [37]	1	1	1	1	0	0	1	0	1	1	1	8
**Elanchezhian 2019** [43]	1	0	0	0	0	0	0	1	0	0	1	3
**Farzamfar 2017** [44]	1	0	0	1	0	0	0	1	1	1	1	6
**Gygax 2011** [46]	1	1	0	1	0	0	1	1	1	1	1	8
**Kara 2020** [38]	1	1	1	1	0	0	1	1	0	1	1	8
**Madbouly 2021** [39]	1	1	1	1	0	0	0	1	1	1	1	8
**Mohamed 2021** [40]	1	1	1	1	0	0	1	1	1	1	1	9
**Mohammed 2022** [41]	1	1	0	1	0	0	0	1	0	1	1	6
**Narimani 2019** [42]	1	1	0	1	0	0	1	1	1	1	1	8
**TOTAL**	11	9	6	9	0	0	6	10	6	9	11	7.0

**Table 3:** MINORS scale scores.

	1. A Clearly Stated Aim	2. Inclusion of Consecutive Patients	3. Prospective Collection of Data	4. Endpoints Appropriate to the Aim of the Study	5. Unbiased Assessment of the Study Endpoint	6. Follow-Up Period Appropriate to the Aim of the Study	7. Loss to Follow Up Less Than 5%	8. Prospective Calculation of the Study Size	9. An Adequate Control Group	10. Contemporary Groups	11. Baseline Equivalence of Groups	12. Adequate Statistical Analyses	TOTAL
**Elanchezhian 2019** [43]	1	0	2	2	0	1	2	0	2	2	2	2	16
**Farzamfar 2017** [44]	2	0	2	1	0	1	0	0	2	2	2	1	13
**TOTAL**	3	0	4	3	0	2	2	0	4	4	4	3	14.5

**Table 4:** Risk of bias.

References	D1	D2	D3	D4	D5	Overall		
**Abdel-Ghafar 2025** [36]	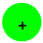	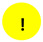	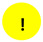	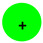	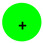	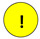	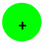	Low risk
**Auld 2017** [45]	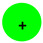	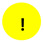	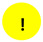	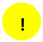	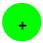	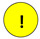	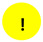	Some concerns
**Bruchez 2016** [37]	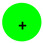	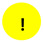	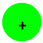	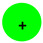	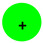	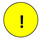	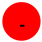	High risk
**Elanchezhian 2019** [43]	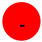	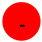	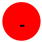	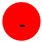	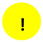	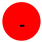		
**Farzamfar 2017** [44]	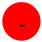	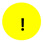	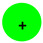	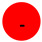	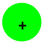	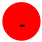	D1	Randomization process
**Gygax 2011** [46]	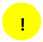	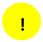	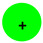	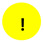	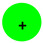	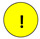	D2	Deviations from the intended interventions
**Kara 2020** [38]	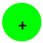	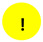	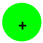	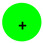	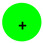	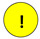	D3	Missing outcome data
**Madbouly 2021** [39]	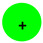	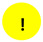	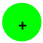	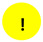	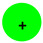	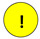	D4	Measurement of the outcome
**Mohamed 2021** [40]	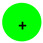	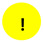	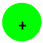	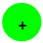	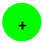	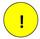	D5	Selection of the reported result
**Mohammed 2022** [41]	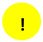	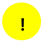	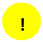	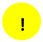	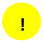	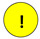		
**Narimani 2019** [42]	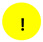	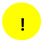	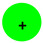	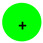	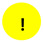	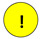		

**Table 5:** Outcome measures and measurement tools.

References	Outcome Measures and Tools																		
	Motor Evaluations													Sensory Evaluations					Others
	BBT	GS	PS	ABILHAND-Kids	QUEST	IS	MAS	FMA-UE	UEFI	Nine-HPT	SHUEE	SCUES	MA2	Tactile Registration	DS	SPL	2PD	Proprioception	COPM
**Abdel-Ghafar 2025** [36]	X	X																	
**Auld 2017** [45]														X	X	X			
**Bruchez 2016** [37]		X	X	X									X				X	X	
**Elanchezhian 2019** [43]							X	X	X	X									
**Farzamfar 2017** [44]	X																		
**Gygax 2011** [46]		X	X								X								
**Kara 2020** [38]					X	X													X
**Madbouly 2021** [39]					X														
**Mohamed 2021** [40]	X	X			X														
**Mohammed 2022** [41]												X							
**Narimani 2019** [42]	X	X																	

2PD: Two-Point Discrimination; BBT: Box and Blocks Test; COPM: Canadian Occupational Performance Measure; DS: Double Simultaneous; FMA-UE: Fugl-Meyer Assessment–Upper Extremity; GS: Grasp Strength; IS: Isometric Strength; MA2: Melbourne Assessment 2; MAS: Modified Ashworth Scale; Nine-HPT: Nine-Hole Peg Test; PS: Pinch Strength; QUEST: Quality of Upper Extremity Skills Test; SCUES: Selective Control of the Upper Extremity Scale; SHUEE: Shriners Hospital Upper Extremity Evaluation; SPL: Single-Point Localization; and UEFI: Upper Extremity Function Index.

## Data Availability

Data sharing is not applicable to this article as no new data were created or analyzed in this study.

## Supplementary Materials

The following supporting information can be downloaded at https://www.mdpi.com/article/10.3390/healthcare13131538/s1, S1: Search strategy; S2: Quality of the reported interventions; and Table S1: Reported interventions (TIDieR checklist).

## Abbreviations

The following abbreviations are used in this manuscript:

MT	Mirror Therapy
UCP	Unilateral Cerebral Palsy
UL	Upper Limb
CP	Cerebral Palsy
TIDieR	Template for Intervention Description and Replication
MACS	Manual Ability Classification System
mCIMT	Modified Constraint-Induced Movement Therapy
RCT	Randomized Controlled Trial
RoB	Risk of Bias
MD	Mean Difference
SD	Standard Deviation
COPM	Canadian Occupational Performance Measure
GS	Grasp Strength
BBT	Box and Blocks Test
QUEST	Quality of Upper Extremity Skills Test
SHUEE	Shriners Hospital Upper Extremity Evaluation
MA2	Melbourne Assessment 2
DS	Double Simultaneous
2PD	Two-Point Discrimination
SPL	Single-Point Localization
