# Supplementary material for: Motor and Sensory Benefits of Mirror Therapy in Children and Adolescents with Unilateral Cerebral Palsy: A Systematic Review and Meta-Analysis
Source: Healthcare (Basel). 2025 Jun 27;13(13):1538. doi: 10.3390/healthcare13131538 (PMC12249144; doi:10.3390/healthcare13131538)
Supplement: Supplementary file 1 [file healthcare-13-01538-s001.zip › healthcare-3683501-supplementary.pdf]

Supplementary Materials

S1. Search strategy

PubMed

Search strategy:

- #1 AND #2 AND #3 [tiab]
- #1 AND #2 [tiab]
- #1 AND #2 [titl]

Population

#1 ("cerebral palsy"[Mesh] OR "unilateral spastic cerebral palsy" OR "hemiplegic cerebral palsy" OR "hemiplegia" OR "unilateral cerebral palsy") NOT "stroke"

Intervention

#2 (mirror therapy"[Mesh] OR "mirror movement therap*[Mesh] OR "mirror box" OR "mirror")

Outcome

#3 (“bimanual performance" OR "somatosensory function" OR "gross motor" OR "fine motor" OR "strength" OR "position" OR "unimanual capacity")

COCHRANE LIBRARY

Search strategy:

- #7 AND #12 [titkwab]
- #7 AND #12 [record title]
- #7 AND #12 AND #20 [titkwab]

Population

- #1 MeSH descriptor: [cerebral palsy] explode all trees
- #2 unilateral spastic cerebral palsy
- #3 hemiplegic cerebral palsy
- #4 hemiplegia
- #5 unilateral cerebral palsy
- #6 MeSH descriptor: [stroke] explode all trees
- #7 #1 AND #2 AND #3 AND #4 AND #5 NOT #6

Intervention

- #8 mirror therapy
- #9 MeSH descriptor: [mirror movement therapy] explode all tree
- #10 mirror box
- #11 mirror
- #12 #8 OR #9 OR #10 OR #11

Outcome

- #13 bimanual performance

- #14 somatosensory function
- #15 gross motor
- #16 strength
- #17 position
- #18 fine motor
- #19 unimanual capacity
- #20 #13 OR #14 OR #15 OR #16 OR #17 OR #18 OR #19

Web of Science

Search strategy: #1 AND #2

Population

#1 (((((TI=(cerebral palsy)) OR TI=(unilateral spastic cerebral palsy)) OR TI=(hemiplegic cerebral palsy)) OR TI=(hemiplegia)) OR TI=(unilateral cerebral palsy)) NOT TI=(stroke)

Intervention

#2 (((TS=(mirror therapy)) OR TS=(mirror movement therapy)) OR TS=(mirror box)) OR TS=(mirror)

LILACS (Virtual Health Library)

Search strategy

- #1 AND #2 [tiabsub]

Population

#1 (unilateral spastic cerebral palsy)

Intervention

#2(mirror therapy)

S2. Quality of the reported interventions

Table S1. Reported interventions (TIDieR checklist)

**References**	**Abdel-Ghafar 2025**	
**Group**	**EG**	**CG**
**Name**	**Mirror Visual Feedback + Standard Rehabilitation Program**	**Modified Constraint-Induced Movement Therapy + Standard Rehabilitation Program**
**Key components**	Mirror Visual Feedback: The following fine motor exercises were carried out: rolling a ball from the tip of the fingers to the palm on a table; moving beads from one cup to another; squeezing a stress ball; stretching rubber bands with fingers; and rotating pencils clockwise and anticlockwise.<br>Standard Rehabilitation Program: functional unilateral upper extremity training and fine motor exercises like cupping the mouth, pulling a circle, and scouring a towel, as well as active wrist and elbow extension, forearm supination, and grasp strength.	mCIMT: Functional tasks with the affected hand, with the unaffected hand restrained by an arm sling during the rehabilitation session. The activities were like dough activities, bottle and marble activities, and manual chores that offered structured and extensive practice with the afflicted hand.<br>Standard Rehabilitation Program: same as EG.
**Materials**	• Mirror<br>• Material for the intervention	• Arm sling<br>• Material for the mCIMT and the Standard Rehabilitation Program
**Provider**	Skilled Physical Therapist	
**When / Setting**	NR	
**Dosage**	Mirror Visual Feedback: 12 weeks, 5 times per week, and the treatment sessions lasted 30 minutes each. Each exercise was performed 10 times, with a rest period of 20 seconds between exercises.<br>Standard Rehabilitation Program: 2 weeks, 3 times per week. The treatment sessions lasted 45 to 60 minutes each.	mCIMT: 12 weeks, 5 times per week, and the treatment sessions lasted 30 min each.<br>Standard Rehabilitation Program: same as EG.
**Tailoring**	NR	Individualized instruction from professionals during the therapy, which included the practice of specific target movements.
**Modifications**	NR	
**Fidelity**	Fidelity checklist was used to ensure adherence to therapy protocols and minimize variability.	
**Compliance**	NR	

References	Auld 2017	
**Group**	**EG**	**CG**
**Name**	Mirror Training	Bimanual Therapy
**Key components**	Alternative motor and tactile components:<br>• Mirror-based tactile training: 3 blocks of 24 tactile stimuli, performed at one out of five possible locations of the affected hand. Stimuli were performed with one larger SWM monofilament that threshold. Each stimulus took 1.5 seconds, with a 20-second rest between stimuli.<br>• Mirror-based movement training: 2 blocks of 15 minutes of copying upper-limb movement positions on verbal command, as provided by the investigator.	Repetitive practice of bimanual age-appropriate activities.
**Materials**	• Mirror box (noigroup.com, Adelaide, Australia)<br>• SWM filaments	NR
**Provider**	Experienced physiotherapist	
**When / Setting**	At the clinic or at the child's home	
**Dosage**	One 90-minute session	One 90-minute session
**Tailoring**	NR	
**Modifications**	NR	
**Fidelity**	NR	
**Compliance**	NR	

References	Bruchez 2016	
Group	EG	CG
Name	**Mirror Therapy**	**Symmetrical and simultaneous exercises without mirror**
Key components	Mirror Therapy exercises: all done with both hands symmetrically.<br>1- Sequential finger-by-finger pressure on modelling-clay<br>2- Thumb–index pinch extension<br>3- Foam palmar squeezing<br>4- Clockwise and anticlockwise rotation of the wrists<br>5- Forearm pronation and supination<br>6- Shoulder antepulsion and retropulsion<br>7- shoulder abduction–adduction<br>+ [usual therapies (occupational and/or physical therapy)]	The same exercises than EG, without the mirror<br>+ [usual therapies (occupational and/or physical therapy]
Materials	• Leaflet and DVD summarizing the instructions and training regimen.<br>• Mirror, size 38x28cm.<br>• Material for the exercises	• Leaflet and DVD summarizing the instructions and training regimen.<br>• Material for the exercises.
Provider	Study coordinator	
When / Setting	At home	
Dosage	15-minute sessions, 5 days a week, for 5 weeks.<br>On each session, participants performed 3x10 repetitions of all exercises.	
Tailoring	NR	
Modifications	NR	
Fidelity	NR	
Compliance	NR	

References	Elanchezhian 2019	
**Group**	**EG**	**CG**
**Name**	**Mirror Therapy**	**Conventional treatment**
**Key components**	Mirror Therapy program:<br>1- Elbow flexion and extension: Moving beads from one cup to another.<br>2- Forearm pronation and supination: Putting pegs in a pegboard<br>3- Wrist flexion and extension: Squeezing a stress ball.<br>4- Wrist ulnar deviation and radial deviation: Stacking pennies.<br>5- Fingers flexion and extension: Rubber band stretching with fingers.<br>6- Finger numbering: Knobs & Screws- clockwise and anti-clockwise.<br>7- Opposition of thumb; clip clothespins or kitchen clips to the edge of a container.<br>8- Spreading of fingers apart; Roll ball from tip of fingers to palm on a table.	Conventional program:<br>1- Passive muscle stretching + wrist splint.<br>2- Encourage the midline following strategies to reach the front of the shoulder.<br>3- Encourage reaching through the use of shoulder flexion and external rotation by putting the object in standing position between the arm and knee based on the child's capacity to regulate external rotation and supination while achieving completion.<br>4- Hand weight bearing at the scapula-humeral area, at the elbow, and wrist. 5- Activities involving touching the desired target and keeping that position for a couple of seconds.
**Materials**	• Mirror, size 30x40<br>• Height-adjustable table<br>• Material for the exercises	• Wrist splint
**Provider**	NR	
**When / Setting**	NR	
**Dosage**	4 weeks (60 min/ each session, 5 days/week)	NR
**Tailoring**	In the event of visuospatial negligence or serious muscle spasticity in the impacted limb, the mirror's location could be adapted so that it pointed more diagonally to the unaffected limb. The image ensured that the mirror image need to match the impacted limb's perception.	NR
**Modifications**	NR	
**Fidelity**	NR	
**Compliance**	NR	

References	Farzamfar 2017	
**Group**	**EG**	**CG**
**Name**	**Mirror Therapy**	**Standard Therapy**
**Key components**	Treatment included different exercises, with a mirror: rotating the device, wheeling shoulder, adduction and abduction of the arm, picking up tennis balls and dropping them in the basket, pushing the cylinder on the table, rolling a tennis and Hedgehog ball on the table, rowing with elastic bands, connecting the dots on the paper, snipping card-board, etc.	The same exercises than EG, without the mirror.
**Materials**	• Adjustable table<br>• Triangular mirror box<br>• Material for the exercises	• Material for the exercises
**Provider**	Therapist	
**When / Setting**	NR	
**Dosage**	16 sessions of 30 minutes each.	
**Tailoring**	NR	
**Modifications**	NR	
**Fidelity**	NR	
**Compliance**	NR	

References	Gygax 2011	
**Group**	**EG**	**CG**
**Name**	**Mirror Therapy**	**Sham condition**
**Key components**	Three repetitive symmetrical upper limb exercises: two with modelling-clay in each hand to elicit bilateral thumb-finger pinch and grasping, and one with a bottle of water to train forearm pro-supination with mirror therapy box.	The same treatment as the EG, without a mirror box.
**Materials**	• Mirror Therapy box<br>• Material for the exercises	• Material for the exercises
**Provider**	NR	
**When / Setting**	NR	
**Dosage**	15 minutes of daily bimanual training with mirror for 3 weeks.	15 minutes of daily bimanual training with mirror for 3 weeks.
**Tailoring**	NR	
**Modifications**	NR	
**Fidelity**	NR	
**Compliance**	NR	

References	Kara 2020	
**Group**	**EG**	**CG**
**Name**	**Mirror Therapy Combined with Strength Exercises**	**Occupational Therapy**
**Key components**	Mirror Therapy exercises:<br>1- Grasping and placing pieces of Cheerios using a bilateral thumb-finger pinch;<br>2- Grasping and placing small pieces in hand exercise putty (CanDo® Theraputty®) using a bilateral thumb-finger pinch<br>3- Grasping and releasing a small ball<br>4- Grasping a bottle of water using forearm pro-supination.<br>Strength exercises:<br>1- Bilateral ball throwing<br>2- Push-ups on stable or unstable surfaces.	The preferred activities involved fine motor skills, such as handwriting, drawing, cutting with scissors, or playing with cubes in services for accessibility, without any mirror.
**Materials**	• Mirror<br>• Material for the exercises	• Material for the exercises
**Provider**	Therapist	
**When / Setting**	NR	
**Dosage**	30-minute sessions, 3 days per week for a period of 12 weeks. The power exercises lasted 30 seconds, during 3 days a week, during 12 weeks	3 days a week for 12 weeks (30 minutes a day).
**Tailoring**	NR	
**Modifications**	NR	
**Fidelity**	NR	
**Compliance**	NR	

References	Madbouly 2021	
Group	GA	GB
Name	**Modified Constraint-Induced Movement Therapy**	**Mirror Therapy**
Key components	Selected physical therapy exercises include:<br>1- Strength exercises for weak wrist extensors, forearm supinator, and for intrinsic muscles of the affected hand.<br>2- Stretching exercises for wrist flexors, fingers flexors and forearm pronators.<br>3- Upper limb weight bearing exercises and overhead activities.<br>4- Grasping and releasing small objects in a container.<br>5- Building a tower with cubes.<br>6- Balance training.<br>+ [regular intensive physical and occupational therapy]	Activities performed by the right hand included grasps of cylindrical using cups, bottle, spherical using plastic balls of various diameter and hook grasp using rings and handles pattern objects. Gripping activities with Theraputty®, pins board for training pincer pattern, attaching and detaching the chains using pulp-to-pulp prehension pattern. Transfer of cubes from one box to other with only left hand.<br>Selected physical therapy exercises include:<br>1- Strength exercises for weak wrist extensors, forearm supinator, and for intrinsic muscles of the affected hand.<br>2- Stretching exercises for wrist flexors, fingers flexors and forearm pronators.<br>3- Upper limb weight bearing exercises and overhead activities.<br>4- Grasping and releasing small objects in a container.<br>5- Building a tower with cubes.<br>6- Balance training.<br>+ [regular intensive physical and occupational therapy]
Materials	• Material for the exercises	• Material for the exercises
Provider	Therapist	
When / Setting	NR	
Dosage	3 hours a day, 5 times per week, for 4 weeks, and regular intensive physical and occupational therapy apart from the therapy.	30 minutes/day, 5 times per week, using a mirror box for 4 weeks and regular intensive physical and occupational therapy apart from the therapy.
Tailoring	NR	
Modifications	NR	
Fidelity	NR	
Compliance	NR	

**References**	**Mohamed 2021**		
**Group**	**GA**	**GB**	**GC**
**Name**	**Mirror Therapy with taping in affected hand**	**Modified Constraint-Induced Movement Therapy**	**Mirror Therapy**
**Key components**	Participants across the three groups followed an identical prescribed upper limb exercise program, with each group receiving different physiotherapy techniques according to their assigned intervention modality. All exercises in the program are described in the paper. In addition, the children in the three groups underwent routine physical therapy programs according to each child's needs (including stretching and strengthening exercises, neurodevelopmental treatment, and balance exercises) for 1 h, which was conducted by a therapist who was not involved in the study.		
**Materials**	• Mirror, 30x20 inches<br>• Kinesio taping (1.5 or 2 inches "I" tape)	• Upper limb sling as the means of restraint.	• Mirror, size 30x20 inches
**Provider**	Researcher		
**When / Setting**	NR		
**Dosage**	1-hour sessions, for 5 days a week, during 12 successive weeks.		
**Tailoring**	NR		
**Modifications**	NR		
**Fidelity**	NR		
**Compliance**	Patients were considered a dropout from the study when more than two sessions were missed, and did not complete >90% of the exercises.		

**References**	**Mohammed 2022**	
**Group**	**EG**	**CG**
**Name**	**Mirror Therapy and neurodevelopment-based guideline program**	**Neurodevelopment-based guideline program**
**Key components**	Mirror Therapy<br>1. Basic exercises: fingers flexion/extension, wrist flexion/extension, ulnar/radial deviation, forearm supination/pronation, elbow flexion/extension, shoulder adduction/abduction)<br>2. Functional tasks: hand grip exercise, putting blocks into bucket, turning cards, moving a small ball, putty palmar squeezing<br>Neurodevelopmental base guideline program (described in control group)	1-Stretching activities for the elbow flexors and forearm supinators of the stretching activities for the elbow flexors and forearm supinators of the upper limbs.<br>2-Fine motor activities.<br>3-Full range of motion tasks.<br>4-Finger/forearm strengthening for the involved side.
**Materials**	• Mirror, size 25 × 20 inches<br>• Table<br>• Chair<br>• Material for the exercises	• Material for the exercises.
**Provider**	NR	
**When / Setting**	NR	
**Dosage**	6 weeks, 3 sessions/week, 60 minutes/session (30 minutes of guideline protocol + 30 minutes of mirror therapy (15' basic exercises + 15' functional tasks)	6 weeks, 3 session/week, 60 minutes/session
**Tailoring**	NR	
**Modifications**	NR	
**Fidelity**	NR	
**Compliance**	NR	

References	Narimani 2019	
Group	EG	CG
Name	**Mirror Therapy + Occupational Therapy**	**Occupational Therapy**
Key components	Mirror Therapy: different exercises, done symmetrically and bimanually, with a mirror. Flexion and extension of the fingers and wrists, supination and pronation of forearm, several functional exercises such as Lego removal, puzzle pieces, circle drawing, squeezing of special balls and towel cloth.<br>Occupational Therapy (described in control group)	Occupational Therapy:<br>1- Several examples of new Bobath exercises: pressure tapping, inhibitory tapping, active, inactive and resistive flexion and extension of the elbow and wrists.<br>2- Several functional exercises: cupping the mouth, pulling a circle, and scouring the towel
Materials	• Mirror, size of 30 × 30 inches<br>• Table<br>• Chair<br>• Material for the exercises	• Material for the exercises
Provider	NR	
When / Setting	NR	
Dosage	3 days/week, 30 min daily, for 6 weeks.<br>Each move was performed 10 times and after about 20 sec of rest, the next move was taken.	NR
Tailoring	NR	
Modifications	NR	
Fidelity	NR	
Compliance	NR	

CG: control group; EG: experimental group; GA: group A; GB: group B; GC: group C; mCIMT: modified Constraint Induced Movement; NR: not reported; SWM: Semmes Weinstein Monofilaments.
